# Overexpression of a pearl millet WRKY transcription factor gene, *PgWRKY74*, in Arabidopsis retards shoot growth under dehydration and salinity-stressed conditions

**DOI:** 10.1007/s10529-024-03492-1

**Published:** 2024-05-08

**Authors:** Maimuna Qazi, Shashi Kumar Gupta, Tetsuo Takano, Daisuke Tsugama

**Affiliations:** 1 Asian Research Center for Bioresource and Environmental Sciences (ARC-BRES), Graduate School of Agricultural and Life Sciences, The University of Tokyo, 1-1-1 Midori-Cho, Nishi-Tokyo-Shi, Tokyo, 188-0002 Japan; 2 International Crops Research Institute for the Semi-Arid Tropics (ICRISAT), Hyderabad, Telangana India

**Keywords:** Arabidopsis, Dehydration, Pearl millet, Salinity, Transcription factor, WRKY

## Abstract

Pearl millet (*Cenchrus americanus*) is a cereal crop that can tolerate high temperatures, drought, and low-fertility conditions where other crops lose productivity. However, genes regulating this ability are largely unknown. Transcription factors (TFs) regulate transcription of their target genes, regulate downstream biological processes, and thus are candidates for regulators of such tolerance of pearl millet. *PgWRKY74* encodes a group IIc WRKY TF in pearl millet and is downregulated by drought. *PgWRKY74* may have a role in drought tolerance. The objective of this study was to gain insights into the physiological and biochemical functions of PgWRKY74. Yeast one-hybrid and gel shift assays were performed to examine transcriptional activation potential and deoxyribonucleic acid (DNA)-binding ability, respectively. Transgenic *Arabidopsis thaliana* plants overexpressing *PgWRKY74-green fluorescent protein* (*GFP*) fusion gene were generated and tested for growth and stress-responsive gene expression under mannitol and NaCl-stressed conditions. A construct with *PgWRKY74* enabled yeast reporter cells to survive on test media in the yeast one-hybrid assays. The electrophoretic mobility of DNA with putative WRKY TF-binding motifs was lower in the presence of a recombinant PgWRKY74 protein than its absence. The *PgWRKY74-GFP*-overexpressing Arabidopsis plants exhibited smaller rosette areas than did wild-type plants under mannitol-stressed and NaCl-stressed conditions, and exhibited weaker expression of *RD29B*, which is induced by the stress-related phytohormone abscisic acid (ABA), under the mannitol-stressed condition.

PgWRKY74 have transcriptional activation potential and DNA-binding ability, and can negatively regulate plant responses to mannitol and NaCl stresses, possibly by decreasing ABA levels or ABA sensitivity.

**Supplementary Information:**

The online version contains supplementary material available at 10.1007/s10529-024-03492-1.

## Introduction

Pearl millet (*Cenchrus americanus*, also known as *Pennisetum glaucum*) is a cereal crop that can thrive under high temperatures, drought, and low-fertility conditions where other crops lose productivity. However, genes regulating this ability are largely unknown. Transcription factors (TFs) regulate transcription of their target genes and thereby regulate downstream biological processes including stress responses. WRKY TFs have the highly conserved WRKY motif that has a role in deoxyribonucleic acid (DNA) binding. It can be classified into three groups (I–III) and eight subgroups (Ia, Ib, IIa–d, IIIa and IIIb) (Wu et al. 2005). Most WRKY TFs studied bind DNA with the W-box TTGAC(C/T) (Eulgem et al. 1999; O’malley et al. 2016) and its variants with (C/T)GAC as the core (Machens et al. 2014). Pearl millet has 97 genes encoding WRKY TFs. Some of these genes are either upregulated or downregulated by drought and salinity stress, raising the possibility that they are involved in regulating pearl millet responses to those stresses (Chanwala et al. 2020). However, functions of pearl millet WRKY TFs are not well studied. *PgWRKY74* (or Pgl_GLEAN_10018194) is a pearl millet gene deduced from the reference genome of pearl millet (Varshney et al. 2017). The *PgWRKY74* sequence can be a part of a larger gene in fact but contains a sequence that encodes a putative DNA-binding domain with the WRKY motif (Chanwala et al. 2020, see also Supplementary Fig. 1a). PgWRKY74 can be classified as a subgroup IIc WRKY TF on the basis of features in its amino acid sequence, and *PgWRKY74* is downregulated by drought stress (Chanwala et al. 2020). In agreement, in a previous transcriptome analysis with RNA sequencing (RNA-Seq), a pearl millet salinity stress-tolerant cultivar, ICMB 01222, expressed *PgWRKY74* more weakly than did a salinity stress-sensitive cultivar, ICMB 081 (Shinde et al. 2018; Qazi and Tsugama 2023; see also Supplementary Table 1). On the basis of the amino acid sequence, PgWRKY74 is expected to be localized in the nucleus (Qu et al. 2023). LxLxL, DLNxxP, LxLxPP, (R/K)LFGV and TLLLFR (where x is any amino acid) are motifs present in many plant transcriptional repressors (Kagale and Rozwadowski 2010), but none of these is present in the PgWRKY74 sequence. PgWRKY74 may therefore be a transcriptional activator. *DREB2A* and *RD29A* are two Arabidopsis genes induced under dehydrating conditions (Yamaguchi-Shinozaki and Shinozaki 1994; Liu et al. 1998), and *RD29B* is an Arabidopsis gene induced either under dehydrating conditions or by a treatment with abscisic acid (ABA), a stress-related phytohormone (Uno et al. 2000). It is possible that PgWRKY74 directly or indirectly regulates expression of pearl millet homologs of *DREB2A*, *RD29A* and *RD29B*. The objective of this study was to gain insights into the physiological and biochemical functions of PgWRKY74. Such insights can help to better understand molecular mechanisms of stress tolerance of pearl millet and to narrow down genes useful for improving it.

Here, *PgWRKY74* is shown to retard plant growth under a mannitol-stressed condition when overexpressed in Arabidopsis. The transcriptional activation potential, DNA-binding ability and subcellular localization of PgWRKY74 are also presented.

## Materials and methods

### Plant materials and growth conditions

The pearl millet drought-tolerant line ICMB 843 was provided by ICRISAT (International Crops Research Institute for the Semi-Arid Tropics, India) and was grown in a growth chamber as described previously (Dudhate et al. 2018). Briefly, its seeds were sown on soil with fertilizers in pots, and the resulting plants were grown under long-day (16-h light/8-h darkness) conditions at 28 °C in the growth chamber. Roots of one-month-old plants were sampled and stored at −80 °C for ribonucleic acid (RNA) isolation followed by complementary DNA (cDNA) synthesis to clone *PgWRKY74* (see the “Arabidopsis transformation and green fluorescent protein (GFP) detection” subsection).

The *Arabidopsis thaliana* ecotype Col-0 was used as the wild-type control for all experiments with Arabidopsis. An Arabidopsis line that has a transfer-DNA (T-DNA) insertion in *WRKY56* (*wrky56* mutant, SAIL_737_D01, see Supplementary Fig. 1a for the T-DNA insertion position) was obtained from the Arabidopsis Biological Resource Center (ABRC, https://abrc.osu.edu/) with the stock number CS876373. Arabidopsis seeds were sterilized with 70% ethanol (v/v) for 3 min and with a sterilization buffer (5% sodium hypochlorite and 0.05% tween 20) for 15 min. Then the seeds were rinsed three times with sterile water and sown on a medium containing half-strength Murashige and Skoog (MS) salts, 1.5% (w/v) sucrose, 0.8% (w/v) agar, 0.5 g/L MES, pH 6.0, and 0.001% (w/v) plant preservative mixture (PPM, Plant Cell Technology, DC). The medium with seeds were incubated at 4 °C for 4 days for stratification, and further incubated at 22 °C under the 16-h light/8-h darkness photoperiod in a growth chamber. To assess root and rosette growth, either mannitol or NaCl was added to the media at the 150 mM or 50 mM final concentration, respectively, and the plates with the media were vertically placed in the growth chamber and incubated as described above. To collect seeds, two-week-old plants were transferred to rockwool cubes and further incubated under the above condition in the growth chamber until they developed mature seeds.

To confirm the T-DNA insertion in *WRKY56*, genomic DNA was extracted from cauline leaves of wild-type and *wrky56* plants as previously described (Tsugama et al. 2018). Polymerase chain reaction (PCR) was run with the resulting DNA as the template, the KOD FX Neo DNA polymerase (Toyobo, Osaka, Japan), the primers listed in Supplementary Table 2 (see also Supplementary Fig. 1a), and the following PCR cycle: 98 °C for 2 min, 35 cycles of (98 °C for 10 s, 58 °C for 20 s, and 72 °C for 1 min), and then 72 °C for 4 min. The resulting PCR products were run on agarose gel and visualized with the Safe Imager blue-light transilluminator (Thermo Fisher Scientific, Waltham, MA) and the Atlas ClearSight (Bioatlas, Tartu, Estonia) fluorescent dye. Plants homozygous for the T-DNA insertion were regarded as the *wrky56* mutants (see Supplementary Fig. 1b for an example of the banding pattern in such a homozygous plant) and used for the above growth test.

### Yeast one-hybrid assays

The coding sequence (CDS) of *PgWRKY74* was amplified by PCR using cDNA (see the “Arabidopsis transformation and green fluorescent protein (GFP) detection” subsection) as template, KOD FX Neo, and the following primer pair: 5′-GGAGAATTCATGCCGTGGACGACGGCCGAGCAG-3′ and 5′-CCCGTCGACTCAAGGACCGAATTGATGCATCCC-3′ (*Eco*RI and *Sal*I sites are underlined, respectively). The resulting PCR product was gel-purified by a FastGene Gel/PCR Extraction Kit, digested by *Eco*RI and *Sal*I, and cloned into the *Eco*RI-*Sal*I site of pGBKT7 (Takara Bio), generating pGBK-PgWRKY74. pGBKT7 and pGBK-PgWRKY74 were transformed into the *Saccharomyces cerevisiae* strain AH109 (Takara Bio) by a lithium acetate method (Gietz et al. 1992). Colonies of transformed yeast cells were obtained on a synthetic dextrose (SD) medium lacking tryptophan (SD/-Trp). Cells from 12 individual colonies for each of pGBKT7 and pGBK-PgWRKY74 were incubated at 28 °C on an SD/-Trp, a low-stringency test medium (SD/-Trp/-His: an SD medium that lacks tryptophan and histidine and that contains 10 mM 3-amino-1,2,4-triazole) and a high-stringency test medium (SD/-Trp/-His/-Ade: an SD medium that lacks tryptophan, histidine and adenine) to examine reporter gene activation.

### Gel shift assays

The CDS of *PgWRKY74* was amplified by PCR using cDNA (see the “Arabidopsis transformation and green fluorescent protein (GFP) detection” subsection) as template, KOD FX Neo, and the following primer pair: 5′-GGACATATGGGTACCATGCCGTGGACGACG-3′ and 5′-CCCGGATCCTCACCATACTAGTAGGACCG-3′ (*Nde*I and *Bam*HI sites are underlined, respectively). The resulting PCR product was gel-purified with a FastGene Gel/PCR Extraction Kit, digested by *Nde*I and *Bam*HI, and cloned into the *Nde*I-*Bam*HI site of pMAL-c5E (New England Biolabs, Ipswich, MA), generating pMAL-c5E-PgWRKY74. pMAL-c5E and pMAL-c5E-PgWRKY74 were transformed into the *Escherichia coli* strain BL21(DE3). Maltose-binding protein (MBP) was induced and purified as previously described (Tsugama et al. 2019). To obtain MBP-fused PgWRKY74 (MBP-PgWRKY74), the *E. coli* cells with pMAL-c5E-PgWRKY74 were cultured at 37 °C in Luria–Bertani medium until the optical density at 600 nm reached 0.5, and incubated at 28 °C for 4 h in the presence of 0.1 mM isopropyl-β-D-thiogalactopyranoside. The cells were then harvested by centrifugation and resuspended in 1 × Tris-buffered saline (TBS, 150 mm NaCl, 20 mm Tris–HCl, pH 7.5) with 1 mg/mL lysozyme. This suspension was frozen at −80 °C and thawed at the room temperature. The freezing and thawing were repeated twice more, and the resulting solution was centrifuged at 12,000 × g for 5 min. MBP-PgWRKY74 in the resulting supernatant was bound to Amylose Resin (New England Biolabs), washed four times with an excess amount of 1 × TBS, and eluted with 20 mM maltose.

Genomic DNA was extracted from the powder of the pearl millet roots (see “Arabidopsis transformation and green fluorescent protein (GFP) detection” subsection) by a DNeasy Plant Mini kit (Qiagen, Hilden, Germany). Promoter sequences shown in Supplementary Table 3 were amplified by PCR using this genomic DNA as the template, KOD FX Neo, the PCR DIG Labeling Mix (Sigma-Aldrich, St. Louis, MO) and 35-b primers that anneal to the ends of those promoter sequences. The resulting PCR products were gel-purified by the FastGene Gel/PCR Extraction Kit, diluted 50 times with distilled water, and used as probes for gel shift assays. This genomic PCR was run, using a regular dNTP mixture instead of the PCR DIG Labeling Mix. The resulting PCR products were gel-purified by the FastGene Gel/PCR Extraction Kit, diluted 50 times with distilled water, and used as competitors for the gel shift assays.

The gel shift assays were performed with the above purified proteins, probes and competitors, essentially as described previously (Tsugama et al. 2012a). Briefly, 20-µL reaction solutions that contained 4% (v/v) glycerol, 60 mM KCl, 10 mM Tris–HCl, pH 7.5, 2 μL of the purified protein solution, 1 μL of the probe solution and 0 or 2 μL of the competitor solution were incubated at the room temperature for 20 min and electrophoresed on a 1% agarose gel in 1 × Tris–acetate/ethylenediaminetetraacetic acid (EDTA) buffer. DNA was transferred from the gel to a Hybond-N^+^ membrane (GE HealthCare, Chicago, IL). The membrane was blocked by 2.5% (w/v) skim milk, reacted with an alkaline phosphatase-conjugated anti-digoxigenin antibody (Sigma-Aldrich) in Tween-TBS (0.1% (v/v) Tween 20 in TBS), washed three times in Tween-TBS, equilibrated in a detection buffer (50 mm NaCl, 50 mm Tris–HCl, pH 9.5), and reacted with CDP-Star (Roche). Resulting chemiluminescent signals were detected by an ImageQuant Las 4000 mini imager (GE HealthCare).

### Arabidopsis transformation and green fluorescent protein (GFP) detection

The roots of one-month-old pearl millet plants (see the “Plant materials and growth conditions” subsection) were frozen in liquid nitrogen and ground with a mortar and a pestle to a fine powder. Total RNA was extracted from this powder by a NucleoSpin RNA Plant kit (Macherey–Nagel, Düren, Germany). cDNA was synthesized from 1 µg of the total RNA with a Prime Script Reverse Transcriptase (Takara Bio, Kusatsu, Japan) and the oligo (dT) primer. The CDS of *PgWRKY74* (Pgl_GLEAN_10018194, Varshney et al. 2017; Chanwala et al. 2020) was amplified by PCR using this cDNA as the template, KOD FX Neo, and the following primer pair: 5′-GGAGGTACCATGCCGTGGACGACGGCCGAGCAGGT-3′ and 5′-CCCACTAGTAGGACCGAATTGATGCATCCCGGCGA-3′ (*Kpn*I and *Spe*I sites are underlined, respectively). The resulting PCR product was gel-purified by a FastGene Gel/PCR Extraction Kit (Nippon Genetics, Bunkyo-ku, Japan), digested by *Kpn*I and *Spe*I, and cloned into the *Kpn*I-*Spe*I site of pBI121-35SMCS-GFP (Tsugama et al. 2012b), generating pBI121-35S-PgWRKY74-GFP. The inserted *PgWRKY74* sequence was confirmed by sequencing. pBI121-35S-PgWRKY74-GFP was transformed into the *Agrobacterium tumefaciens* strain EHA105. The resulting *Agrobacterium* cells were used to transform Arabidopsis as previously described (Clough and Bent 1998). Transformed plants were selected by kanamycin in the T1 generation. GFP signals in roots of seedlings in the T2 generation were detected by fluorescence microscopy as previously described (Tsugama et al. 2019). Only plants with the GFP signals were regarded as the *PgWRKY74-GFP*-overexpressing (PgWRKY74-GFPox) plants in the root growth test (see “Plant materials and growth conditions” subsection).

### Quantitative reverse transcription-PCR (qRT-PCR)

Twelve-day-old Arabidopsis seedlings grown in the presence of 0 or 150 mM mannitol (see “Plant materials and growth conditions” subsection) were frozen in liquid nitrogen and ground with a mortar and a pestle to a fine powder. Total RNA was extracted from this powder by NucleoSpin RNA Plant. cDNA was synthesized from 1 µg of the total RNA with a Prime Script Reverse Transcriptase and the oligo (dT) primer. Quantitative PCR (qPCR) was run with the resulting cDNA solutions as templates, the StepOne Real-Time PCR System (Thermo Fisher Scientific), TB Green Premix Ex Taq (Takara Bio), primers listed in Supplementary Table 4 and the following PCR cycle: 95 °C for 30 s, 40 cycles of (95 °C for 5 s and 60 °C for 30 s). Relative expression levels were obtained by the comparative threshold cycle (C_T_) method using the *GAPDH* gene as the internal control. For each plant line, a set of the above experiments (i.e., sampling, RNA extraction, cDNA synthesis and qPCR) was performed three times independently, and the resulting data were regarded as three biological replicates. Three technical replicates were made for each biological replicate in the qPCR.

### RNA-Seq data analysis

Reads derived from previous RNA-Seq with salinity-stressed leaves of pearl millet (Shinde et al. 2018) were downloaded from the sequence read archives (SRA) of the National Center for Biotechnology Information (NCBI, https://www.ncbi.nlm.nih.gov/sra) with the accession number SRP128956. These reads were mapped to the reference genome sequence of pearl millet (Varshney et al. 2017) by the BWA (Burrows-Wheeler Alignment)-MEM software, which can split and align both short and long reads to a reference sequence (Li and Durbin 2010). Read counts for each gene (or transcript) of pearl millet were obtained by featureCounts (Liao et al. 2014). One replicate per sample was available for the data from SRP128956, and the genes where absolute read counts were more than 10 in any sample and where the read counts in one sample were two or more times as great as those in another sample were regarded as differentially expressed genes (DEGs). Five thousand-kb sequences of pearl millet gene promoters were obtained from TGIF-DB (Terse Genomics Interface for Developing Botany, https://webpark2116.sakura.ne.jp/rlgpr), which is a database of genes of pearl millet and other crops (Tsugama and Takano 2021), and converted to either 500-b or 1000-b promoter sequences by a custom Perl script. Motifs enriched in the 500-b or 1000-b promoter sequences of the above DEGs were detected by Homer (Heinz et al. 2010). The data resulting from these analyses were deposited in the figshare repository (Qazi and Tsugama 2023). The custom script used for these analyses can be provided upon request.

### Accession numbers

Details about the sequences of the genes used are available with the Arabidopsis Genome Initiative (AGI) accession number AT1G64000 for *WRKY56*, AT5G52310 for *RD29A*, AT5G05410 for *DREB2A*, AT5G52300 for *RD29B*, with the International Pearl Millet Genome Sequencing Consortium (IPMGSC) accession number Pgl_GLEAN_10018194 for *PgWRKY74* deduced from the pearl millet reference genome, and with the NCBI GenBank accession number OR763013 for the *PgWRKY74* CDS cloned from ICMB 843.

## Results

### PgWRKY74 has transcriptional activation potential and DNA-binding ability

The *PgWRKY74* CDS deduced from the pearl millet reference genome (Varshney et al. 2017; Chanwala et al. 2020) was compared with the *PgWRKY74* CDS cloned from the drought-tolerant pearl millet line ICMB 843. Single-nucleotide substitutions were identified at the nucleotide positions 63 and 108, but the amino acid sequences deduced from those CDSs were identical (Supplementary Fig. 2).

**Fig. 1:**
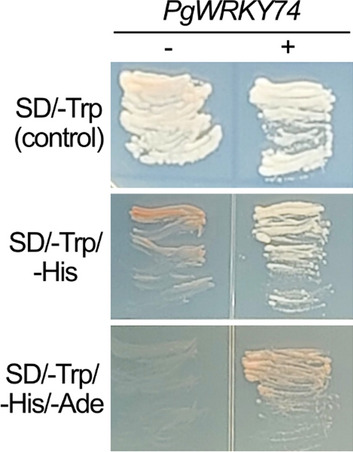
*PgWRKY74* can activate reporter genes in a yeast one-hybrid system. A construct with or without *PgWRKY74* (PgWRKY74 + or −, respectively) was introduced into a yeast reporter strain. For each construct, 12 individual colonies were grown on the media indicated in the figure, and representative images are presented

In a yeast one-hybrid system, the yeast cells transformed with *PgWRKY74* grew faster on test selection media, SD/-Trp/-His and SD/-Trp/-His/-Ade, than those without *PgWRKY74* (Fig. 1). These results suggest that PgWRKY74 has transcriptional activation potential.

**Fig. 2:**
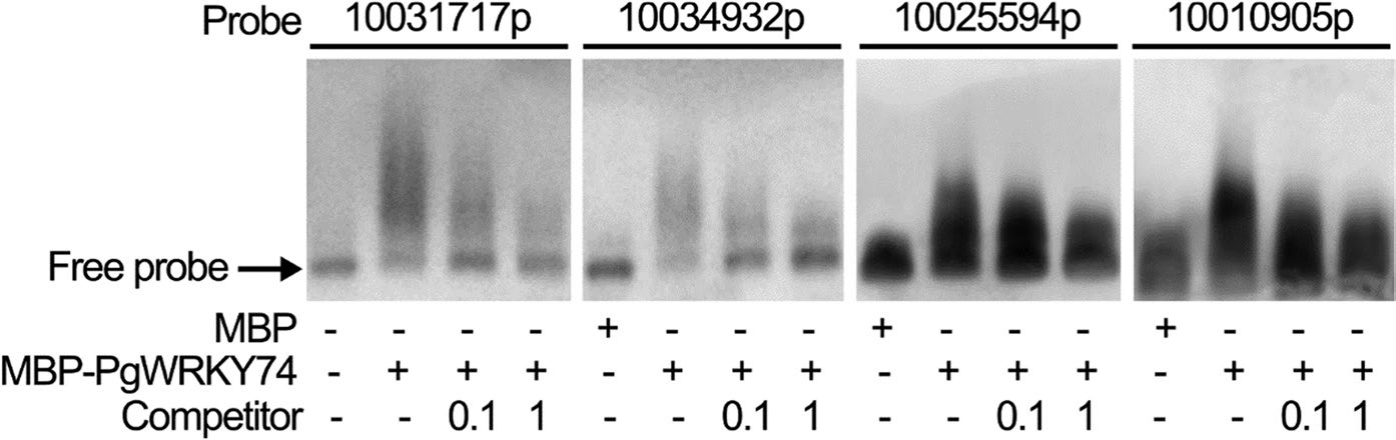
PgWRKY74 binds DNA in vitro. Four DIG-labeled probes indicated in the top of the figure (see also Supplementary Table 3) were used for the analysis. Unlabeled DNA with those sequences were used as competitors. The presence and absence of MBP, MBP-PgWRKY74 and the competitors in the reaction mixtures are indicated by + and −, respectively, in the bottom of the figure. For each probe, experiments were performed three times, and a representative image is presented

The electrophoretic mobility of four probes with the potential WRKY TF-binding sequence (C/T)GAC (see Supplementary Table 3 for these probes) was all lower in the presence of a purified form of MBP-PgWRKY74 than in its absence. The MBP-PgWRKY74-dependent shifts of the electrophoretic mobility of the probes were attenuated by competitors (i.e., DNA fragments that should compete with the probes for MBP-PgWRKY74) (Fig. 2). These results suggest that PgWRKY74 can bind such DNA in vitro.

### Overexpression of *PgWRKY74* in Arabidopsis retards growth under mannitol-stressed conditions

**Fig. 3:**
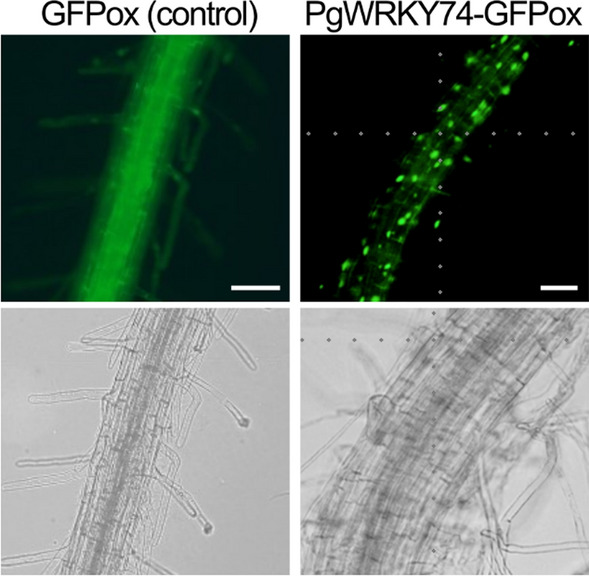
PgWRKY74 is localized in the nucleus. GFP signals in roots of *GFP*-overexpressing (GFPox) and *PgWRKY74-GFP*-overexpressing (PgWRKY74-GFPox) plants were detected by fluorescence microscopy. For each of these proteins, at least three individual plants were used for the signal detection, and a representative image is presented. Scale bars = 50 µm

Arabidopsis lines overexpressing a *GFP*-fused form of *PgWRKY74* under the control of the 35S promoter (PgWRKY74-GFPox) were generated. PgWRKY74-GFP signals in root cells were detected as a single dot (Fig. 3), suggesting that PgWRKY74 is localized in the nucleus.

**Fig. 4:**
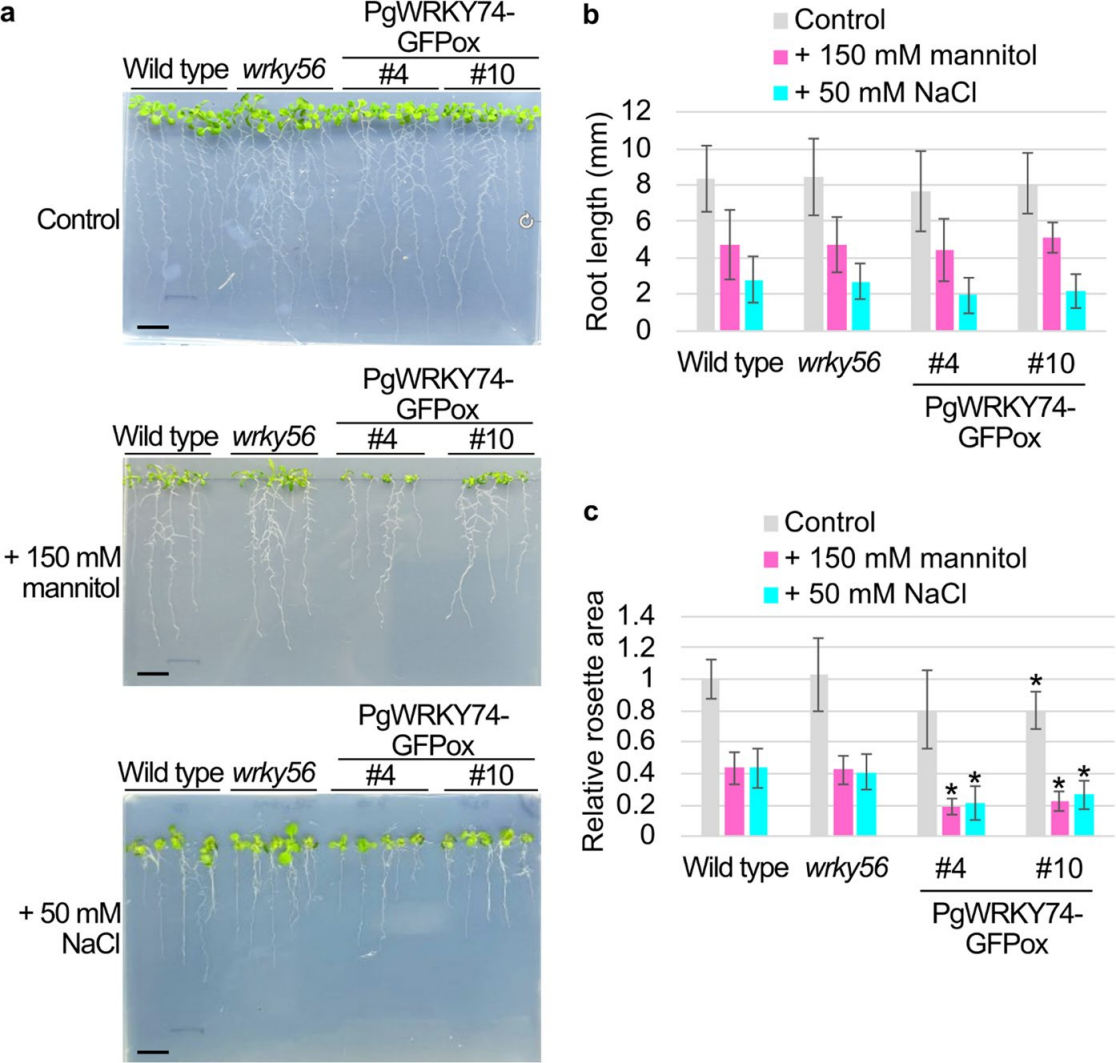
*PgWRKY74-GFP*-overexpressing (PgWRKY74-GFPox) plants exhibit rosette growth retardation under mannitol- and NaCl-stressed conditions. **a** Images of 10-day-old plants grown in the presence of 150 mM mannitol (middle panel) or 50 mM NaCl (bottom) or in their absence (top). Scale bars = 1.5 cm. **b** Root length of the 10-day-old plants. Data are means ± SD (n = 15). *: *P* < 0.001 in Student’s *t*-test vs. the data for the wild type. **c** Relative rosette areas of the 10-day-old plants. Data are means ± SD (n = 15). *: *P* < 0.001 in Student’s *t*-test vs. data for the wild type

The Arabidopsis *wrky56* mutant has a T-DNA insertion in *WRKY56* (Supplementary Fig. 1), one of the closest *PgWRKY74* homologs in Arabidopsis, and can be deficient in *WRKY56* functions. Four PgWRKY74-GFPox lines (#4, #10, #17 and #24), which exhibited strong *PgWRKY74* expression (Supplementary Fig. 3), and *wrky56* plants were tested for growth under mannitol (150 mM)- and NaCl (50 mM)-stressed conditions. No difference was observed in growth under these conditions between wild-type and the *wrky56* plants. In contrast, under the mannitol-stressed condition, all of the four PgWRKY74-GFPox lines exhibited smaller rosette areas than did the wild type. Under the NaCl-stressed condition, the PgWRKY74-GFPox lines #4, #10 and #17 exhibited smaller rosette areas than did the wild type (Fig. 4 and Supplementary Fig. 4). These results suggest that the overexpression of *PgWRKY74-GFP* retards shoot growth under these stressed conditions in Arabidopsis.

**Fig. 5:**
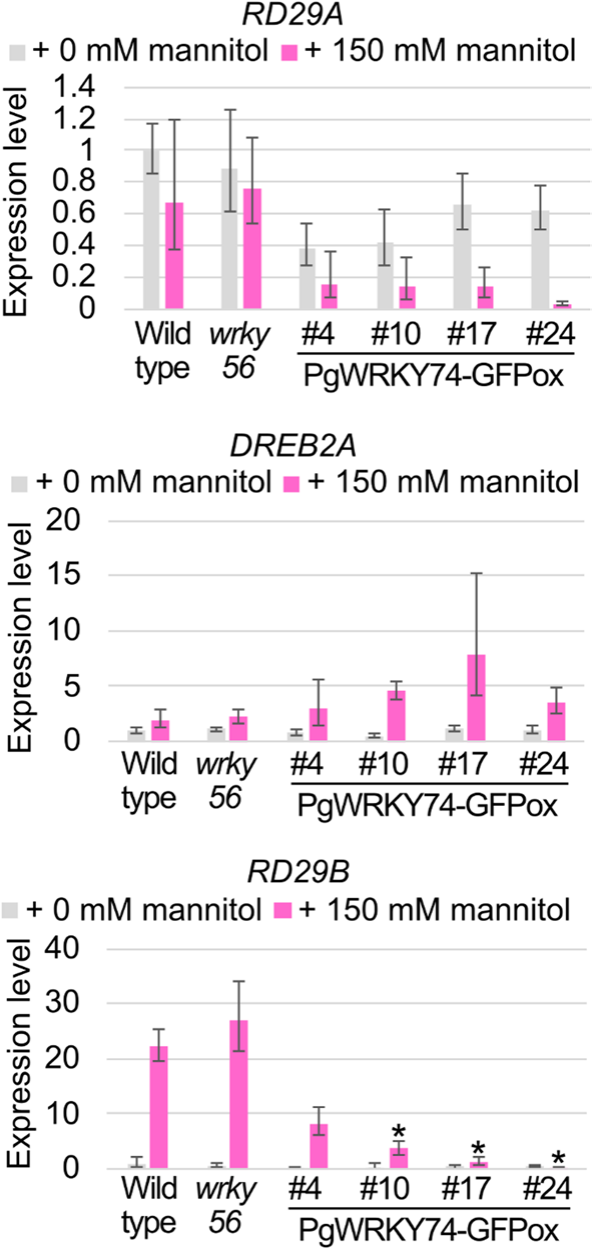
*PgWRKY74-GFP*-overexpressing (PgWRKY74-GFPox) plants exhibit decreased expression of *RD29B* under a mannitol-stressed condition. Twelve-day-old PgWRKY74-GFPox seedlings were subjected to the RNA extraction followed by the cDNA synthesis and PCR. The relative expression levels were obtained by the comparative threshold cycle (C_T_) method using the *GAPDH* as the internal control gene. *: *P* < 0.005 in Student’s *t*-test vs. data for the wild type

No difference was observed between the wild type and any of the PgWRKY74-GFPox lines in expression of *RD29A* or *DREB2A* under either the mannitol-stressed or an unstressed condition (Fig. 5, top and middle panels). However, *RD29B* expression was weaker in the PgWRKY74-GFPox lines #10, #17 and #24 than the wild type (Fig. 5, bottom). These results raise the possibility that the *PgWRKY74-GFP* overexpression negatively regulates ABA responses in Arabidopsis.

### Potential involvement of *PgWRKY74* in the difference in salinity stress tolerance between pearl millet cultivars

In both 500-b and 1000-b promoters of the genes that were expressed more weakly in ICMB 01222 than in ICMB 081 under the salinity-stressed condition, a motif containing a W-box was found to be enriched (Supplementary Fig. 5). These results raise the possibility that a WRKY TF is involved in the difference in salinity stress tolerance between those cultivars and that PgWRKY74 is candidate for such a WRKY TF.

## Discussion

The data presented in this study suggest that PgWRKY74 exhibits transcriptional activation potential in a yeast one-hybrid system, exhibits DNA-binding ability in vitro and is localized in the nucleus. These findings support the idea that PgWRKY74 functions as a transcription factor.

PgWRKY74 is a group II (subgroup IIc) WRKY TF (Chanwala et al. 2020) and could interact with two probes that contained (C/T)GAC but lacked a W-box (TTGAC(C/T)) (probes “10025594p” and “10010905p”, Fig. 2). (C/T)GAC was originally identified as a core of a non-W-box sequence bound by WRKY70, a group III WRKY TF in Arabidopsis (Machens et al. 2014). These findings raise the possibility that not only group III WRKY TFs but also at least some group II WRKY TFs can bind to a non-W-box sequence with (C/T)GAC.

*PgWRKY74-GFP* overexpression in Arabidopsis retarded rosette growth under mannitol- and NaCl-stressed conditions, and decreased the expression of the ABA-inducible gene *RD29B* under the mannitol-stressed condition. In a previous study, overexpression of *GhWRKY68*, a group IIc WRKY TF gene of cotton (*Gossypium hirsutum*), in tobacco (*Nicotiana benthamiana*) retarded plant growth under mannitol-stressed and NaCl-stressed conditions, decreased expression of stress-responsive genes under those conditions, decreased the extent of ABA-induced stomatal closure, and decreased ABA levels in seedlings (Jia et al. 2015). These findings are consistent with each other and support the idea that at least some group IIc WRKY TFs negatively regulate ABA levels and/or responses to dehydration and salinity stress. The drought- and salinity stress-induced downregulation of *PgWRKY74* (Shinde et al. 2018; Chanwala et al. 2020) may therefore contribute to increasing ABA levels and/or drought responses in pearl millet under a drought-stressed condition. On the other hand, overexpression of *HbWRKY82*, a group IIc WRKY TF gene of the Pará rubber tree (*Hevea brasiliensis*), in Arabidopsis promoted plant growth under mannitol-stressed and NaCl-stressed conditions, although it decreased the expression of *RD29B* under those conditions (Kang et al. 2021) as did overexpression of *PgWRKY74-GFP*. This raises the possibility that some group IIc WRKY TFs positively regulate responses to dehydration and salinity stress. It will be interesting to study roles of other group IIc WRKY TFs in responses to dehydration and salinity stress in pearl millet. This can contribute to narrowing down the WRKY TFs relevant to differences in such responses between pearl millet cultivars. Two Arabidopsis group IIc WRKY TFs, WRKY50 and WRKY51, regulate plant responses to another stress-related phytohormone, jasmonic acid (Gao et al. 2011). Another Arabidopsis group IIc WRKY TF, WRKY71, regulates flowering (Yu et al. 2016) and leaf senescence (Yu et al. 2021). It will also be interesting to determine whether PgWRKY74 and other group IIc WRKY TFs in pearl millet have such functions.

## Supplementary Information

Below is the link to the electronic supplementary material.

Supplementary file1 (PDF 654 KB)

## Data Availability

The datasets generated during and/or analyzed during the current study are available in the figshare repository, https://doi.org/10.6084/m9.figshare.24531097 (Qazi and Tsugama 2023).
