# Supplementary material for: Overexpression of a pearl millet WRKY transcription factor gene, PgWRKY74, in Arabidopsis retards shoot growth under dehydration and salinity-stressed conditions
Source: Biotechnol Lett. 2024 May 8;46(5):851–60. doi: 10.1007/s10529-024-03492-1 (PMC11415432; doi:10.1007/s10529-024-03492-1)
Supplement: Supplementary file 1 — Supplementary file1 (PDF 654 KB) [file 10529_2024_3492_MOESM1_ESM.pdf]

Supplementary data for

Overexpression of a pearl millet WRKY transcription factor gene, *PgWRKY74*, in Arabidopsis retards shoot growth under dehydration and salinity-stressed conditions

Maimuna Qazi[1], Shashi Kumar Gupta[2], Tetsuo Takano[1], Daisuke Tsugama[1]*

[1]Asian Research Center for Bioresource and Environmental Sciences (ARC-BRES), Graduate School of Agricultural and Life Sciences, The University of Tokyo, 1-1-1 Midori-cho, Nishitokyo-shi, Tokyo 188-0002, JAPAN.

[2]International Crops Research Institute for the Semi-Arid Tropics (ICRISAT), Hyderabad, Telangana State, India.

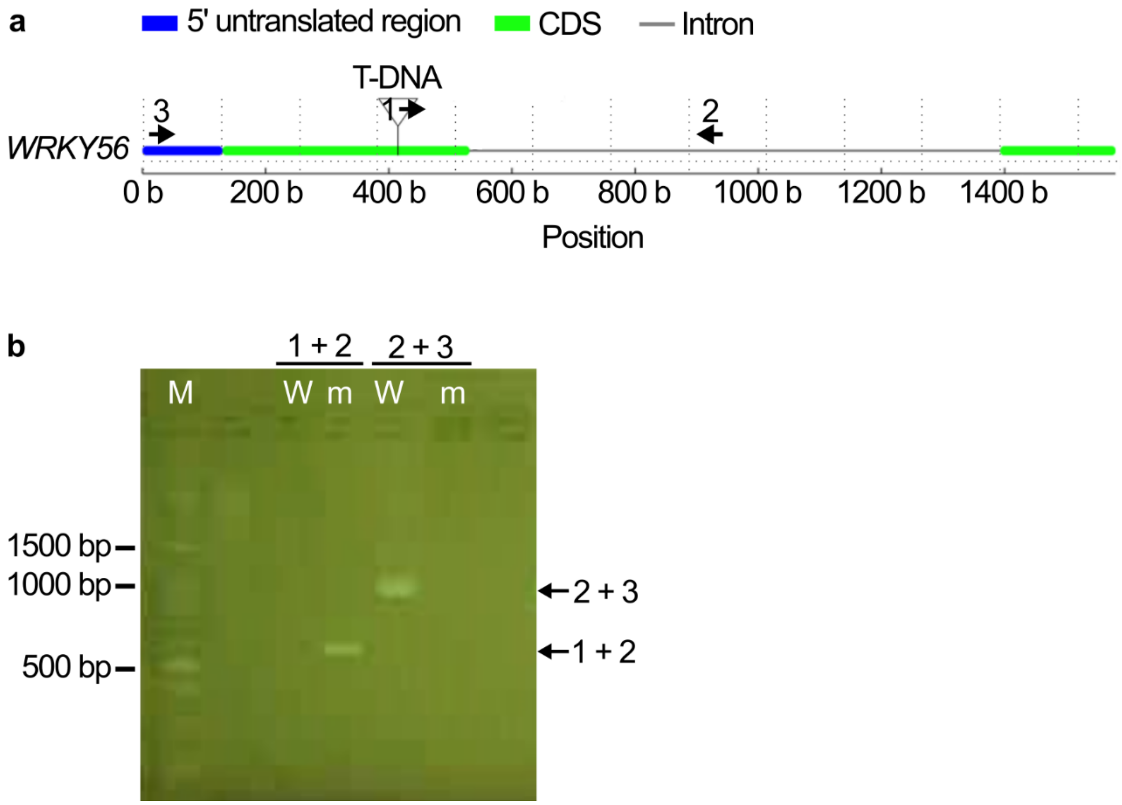

Supplementary Fig. 1. The T-DNA insertion in the *wrky56* mutant. (a) Schematic representation of the *WRKY56* (AT1G64000) and the T-DNA insertion in this locus. Annealing site for the primers used to detect the T-DNA insertion are indicated by arrows. The numbers for these primers correspond to the numbers in the panel b. (b) Genomic PCR analysis of the T-DNA insertion. Genomic DNA was prepared from a wild-type plant (W) and a plant homozygous for the T-DNA insertion in *WRKY56* (m), and used as templates for the PCR. The primer combinations and the positions of the resulting PCR products are indicated in the top and the right side of the figure. M: DNA size marker. Signals are expected to be obtained from the primer pair "1 + 2" if it is used with genomic DNA from plants either homozygous or heterozygous for the T-DNA insertion. So are signals from the primer pair "2 + 3" if it is used with genomic DNA from plants either heterozygous for the T-DNA insertion or without the T-DNA insertion.

a

>Pgl_GLEAN_10018194_CDS (411 bases)
ATGCCGTGGACGACGGCCGAGCAGGTCGTCGTGCCGGACGCCTCGGGCTACGCTCAT
GCTCGTGGTGTGGCCGCCGTGGTCGGGGAGGGGACGACGGCGAGGACGACGGACA
GCAGGATCGCGTTCCGAGTGAGGTCGGAGGAGGAGGTACTCGATGACGGCTACAAG
TGGAGGAAGTACGGCAAGAAGTCCGTCAAGAACAGCCCTAATCCGAGGAACTATTAC
CGGTGCTCGACGGAGGGCTGCAGCGTCAAGAAGAGGGTAGAGCGAGACAAGGACG
ACCCGAGCTACGTTGTGACCATGTACGAGGGGGTGCACAATCACGTTAGCCCCGGCA
CCGTCTACTACGCCACCCAAGACGCCGCCTCTGGCCGCTTCTTTGTCGCCGGGATGCA
TCAATTCGGTCCTTGA

> OR763013 (411 bases)
ATGCCGTGGACGACGGCCGAGCAGGTCGTCGTGCCGGACGCCTCGGGCTACGCTCAT
GCTCGGGGTGTGGCCGCCGTGGTCGGGGAGGGGACGACGGCGAGGACGACTGACA
GCAGGATCGCGTTCCGAGTGAGGTCGGAGGAGGAGGTACTCGATGACGGCTACAAG
TGGAGGAAGTACGGCAAGAAGTCCGTCAAGAACAGCCCTAATCCGAGGAACTATTAC
CGGTGCTCGACGGAGGGCTGCAGCGTCAAGAAGAGGGTAGAGCGAGACAAGGACG
ACCCGAGCTACGTTGTGACCATGTACGAGGGGGTGCACAATCACGTTAGCCCCGGCA
CCGTCTACTACGCCACCCAAGACGCCGCCTCTGGCCGCTTCTTTGTCGCCGGGATGCA
TCAATTCGGTCCTTGA

b

>Pgl_GLEAN_10018194_ OR763013_protein (136 amino acids)
MPWTTAEQVVVPDASGYAHARGVAAVVGEGTTARTTDSRIAFRVRSEEEV**LDDGYKW
RKYGKKSVKNSPNPRNYYRCSTEGCSVKKRVERDKDDPSYVVTMYEGVHNH**VSPG
TVYYATQDAASGRFFVAGMHQFGP

Supplementary Fig. 2. The CDSs of *PgWRKY74* and amino acid sequence deduced from them.

(a) The CDSs of *PgWRKY74*. The top sequence is the *PgWRKY74* CDS deduced from the pearl millet reference genome (Varshney et al. 2017, Pgl_GLEAN_10018194). The bottom sequence is the *PgWRKY74* CDS cloned from ICMB 843 in this study (GenBank accession number: OR763013). Two single-nucleotide substitutions at the positions 63 and 108 are shown in red and underlined. (b) The amino acid sequence of PgWRKY74 deduced from the CDSs. The 51st-108th amino acids correspond to the putative WRKY DNA-binding domain and is underlined. The WRKY motif in this domain is highlighted.

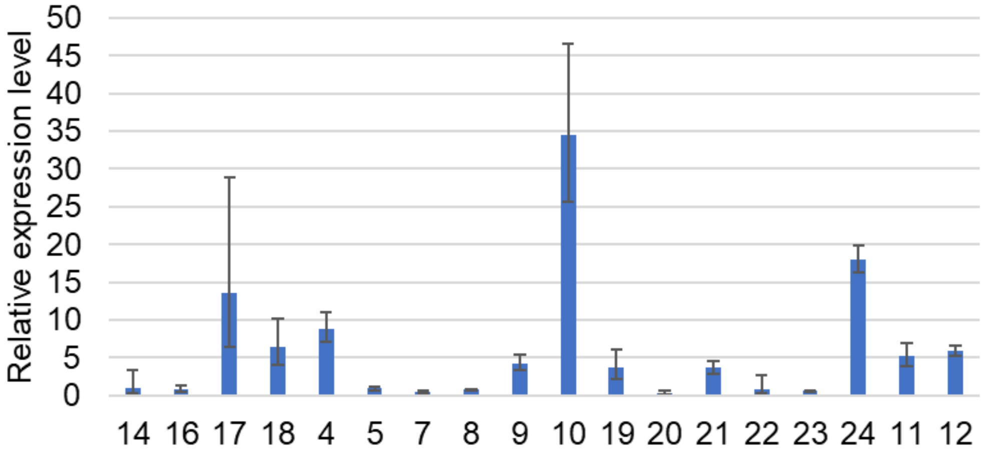

Supplementary Fig. 3. Expression levels of *PgWRKY74* in the Arabidopsis PgWRKY74-GFPox lines. Twelve-day-old seedlings of the PgWRKY74-GFPox lines indicated in the x axis were subjected to the RNA extraction followed by the cDNA synthesis and PCR. The relative expression levels were obtained by the comparative threshold cycle (C_T) method using the *GAPDH* as the internal control gene. Data are means ± SD from three biological replicates.

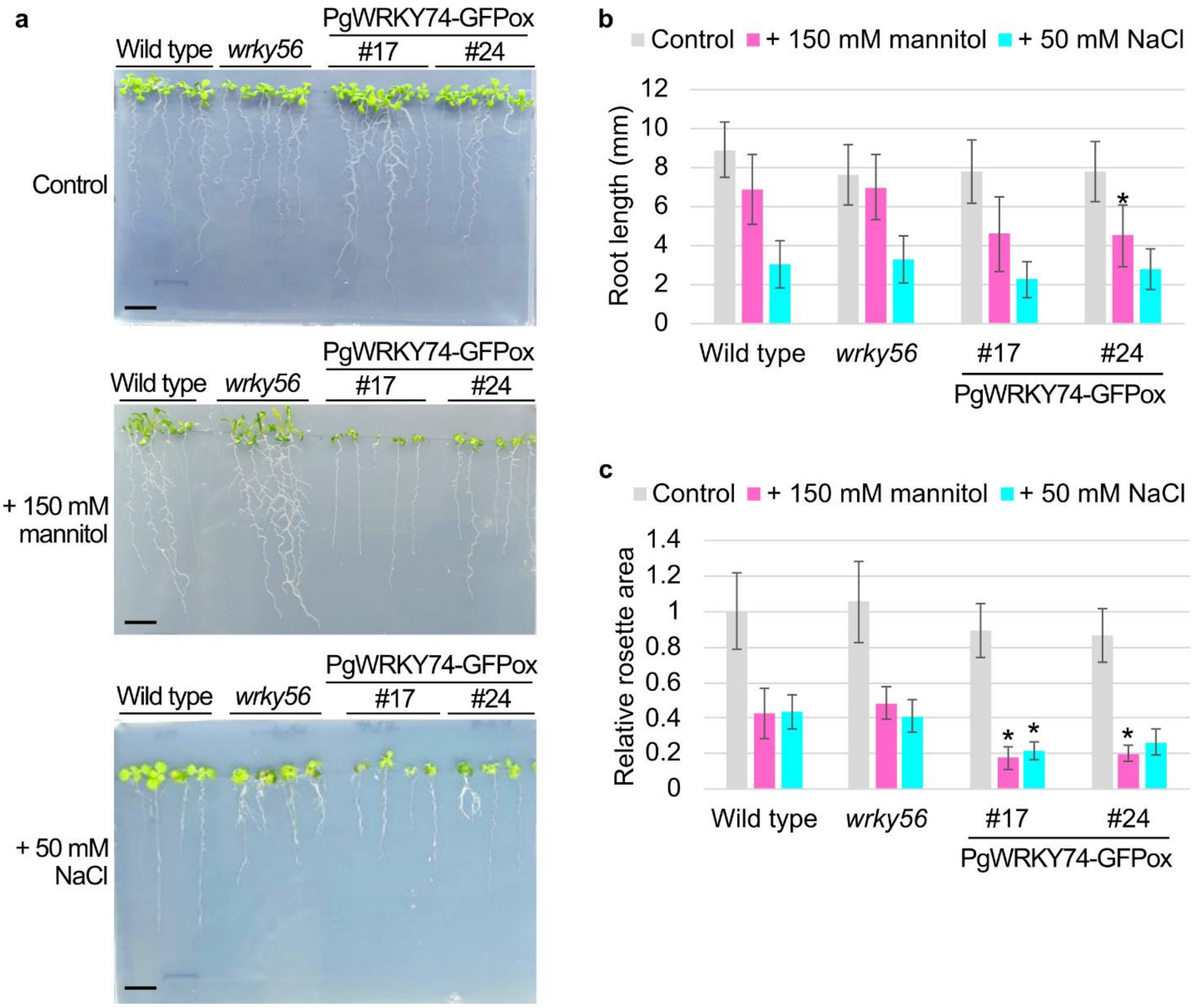

Supplementary Fig. 4. Phenotypes of the PgWRKY74-GFPox lines #17 and #24 under mannitol- and NaCl-stressed conditions. (a) Images of 10-day-old plants grown in the presence of 150 mM mannitol (middle panel) or 50 mM NaCl (bottom) or in their absence (top). Scale bars = 1.5 cm. (b) Root length of the 10-day-old plants. Data are means ± SD (n = 15 for the control and the 150 mM mannitol-stressed conditions; n = 12 for the 50 mM NaCl-stressed condition). *: $P < 0.001$ in Student's *t*-test vs. the data for the wild type. (c) Relative rosette area of the 10-day-old plants. Data are means ± SD (n = 15 for the control and the 150 mM mannitol-stressed conditions; n = 12 for the 50 mM NaCl-stressed condition). *: $P < 0.001$ in Student's *t*-test vs. data for the wild type.

a

* - possible false positive

Rank	Motif	P-value	log P-pvalue	% of Targets	% of Background
1	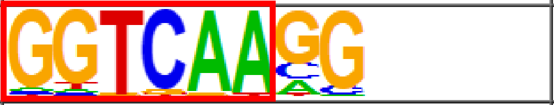	1e-12	-2.973e+01	25.63%	18.65%
2	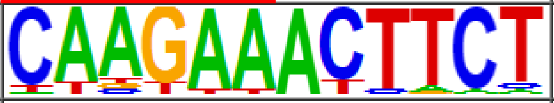	1e-12	-2.768e+01	1.05%	0.00%
3 *	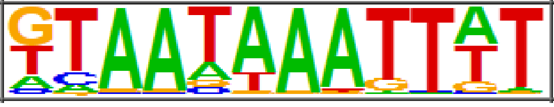	1e-11	-2.693e+01	3.46%	1.13%

b

* - possible false positive

Rank	Motif	P-value	log P-pvalue	% of Targets	% of Background
1	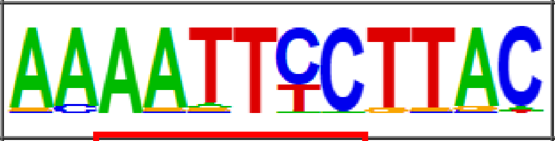	1e-13	-3.187e+01	1.20%	0.03%
2	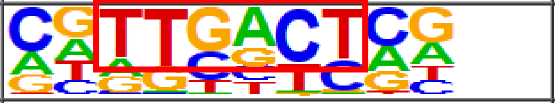	1e-13	-3.043e+01	13.34%	8.10%
3	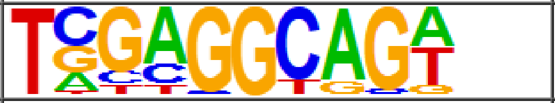	1e-12	-2.925e+01	6.31%	2.84%
4	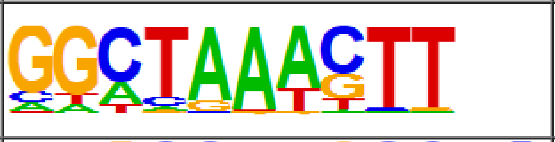	1e-12	-2.854e+01	7.40%	3.68%
5 *	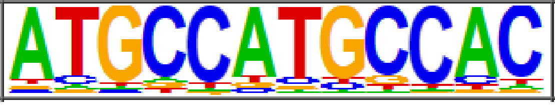	1e-11	-2.581e+01	2.88%	0.83%

Supplementary Fig. 5. Motifs enriched in the promoters of genes that are more weakly expressed in a salinity stress-tolerant line, ICMB 01222, than in a salinity stress-sensitive line, ICMB 081. (a) Such motifs detected in 500-b promoter sequences. (b) Such motifs in 1000-b promoter sequences. For both panels A and B, the W-box (TTGAC(C/T) or its complementary sequence (A/G)GTCAA) is boxed.

Supplementary Table 1. Counts of RNA-Seq derived reads mapped to *PgWRKY74*.

**Sample**	**Read count**
Salinity-stressed ICMB 081 (salinity stress-sensitive)	940
Salinity-stressed ICMB 01222 (salinity stress-tolerant)	427
Unstressed ICMB 081 (salinity stress-sensitive)	1429
Unstressed ICMB 01222 (salinity stress-tolerant)	444

Supplementary Table 2. Primers used to identify the *wrky56* mutant.

**Primer name (Name in Supplementary Fig. 1A)**	**Sequence (5’ to 3’)**
PDAB101LB1 (1)	GCCTTTTCAGAAATGGATAAATAGCCTTGCTTCC
CS876373_LP (2)	GCAACAACCAAACCATATTCG
CS876373_RP (3)	TTCTCCTCTTCTCCTTCTTCCTC

Supplementary Table 3. Promoters used to generate probes for gel shift assays.

Gene for the promoter (probe name in Fig. 2)	Short description based on an Arabidopsis homolog	Promoter sequences used[a]
Pgl_GLEAN_ 10031717 ("10031717p")	ZIM motif family protein Jasmonate ZIM-domain protein 9, TIFY family, previously known as ZIM motif family protein PgTIFY01 (TIFY superfamily-cl5618,PHA03269),short possible domain that contain GATA domain as well as other motifs. Jasmonic acis related stress response	GCACTCGCTGACTTGGTGGACCCGCAGACACGCGGACCCCGCGTGTCATAGGGGTGCCCGGGTGGAGGCGTGGTGGGGGCGCGTCGGGCGCCCATGTGGGCGGACCACGTGGGGGTCCTCTCGCTCGCCTGTAGCCGGTAGCCGGTCGCCGCGGCGGGGCATTAAGTGGCTAATCATCGCCGCTTTCTTCCTCCACCACCGCCTCCCTCCTCCTAAATTCTCGAGGTTAAATAAGCGAGCTGGAGAAAGCAGTTCGGAGCCTAGTCTAGTGAGAAAGGGAGAGGAGGGAGGAAAGGGAAAGCGGCAGGGAGCAAAGAAGAAAGGTCAAGGCATCTGGGGTGCTTCTTCTTCTTCTCCTTCTTTCCGGAGATTCTTGGGACGAGGCGCCGCGGAAGGGAATTTCTCTGGCCTCCGCCGCCACAGCTTCC
Pgl_GLEAN_ 10034932 ("10034932p")	Exostosin family protein GUT2; FUNCTIONS IN: glucuronoxylan glucuronosyltransferase activity	CCTTTTCGTAATCCACGAACCAACTAGCTTGTAGCTTCTGCATTCAGTCAATTGCTCTTTGCTCAGCGACACGGAGTCAGCGTGCAGGATGAATCTCGTCCTATAAAGCGACGTGATTGCCAGTGCTCCTAAGTCTCGGGATCAATCGAACCTGTTATTATTACTATGGCATGATGCATCTGCAGTGGATTATGGTAGATCCTTCCTTCATTCAATAAGTCACTTGTTTTAGTCTCCTTCATCCTTTCCATAGTTGCTAAATTTAGTCCATACCTATTTGGTTCAAAAGATTCATAGGGACCACAACAAATGAGAGGGCATCTCTCTCCATTTAGTCCCAATAAGCAACCCTTTGGTGACTTGTTGGCTTATGAATTGAGACTAAATTTAATCCTCATTAAGTCACCATGTTTGGCACCAAAGTGACTTAATGGAACTTAATTTAGGTTGGCTAGAATAAGTAACCCCAATCCAAGCAGGGCCTGAGCTGCTGAGCATAA
Pgl_GLEAN_ 10025594 ("10025594p")	Xanthine/uracil permease family protein Xanthine/uracil permease family protein; FUNCTIONS IN: transmembrane transporter activity	CAGAGCGGATCTGAGGGCATTTCGCAGCATTCAGGAGCGCTTTTGGGGATTTCTCAGCGCATCCTGTAGCATTTCTCAACGATTCGGTGAGCATTTCGTGCCTTTCTTTTCCCGGTCCCGTGGGTAATTACGTGTTGTTAAGTTACTCGTCGTTGAGGGTTTAATGTCCACTCTGTTGTGATTCAGGCTCGTCAAGTTCTTGATCGTTGGTTAAACATGTTTTCGAGTTCTAATTCCATGTTTAGAGAGGGATTAGGATTGTTTTGCAACCCTTCCAGATTGTTGTACATCTGAAAGTTCTCGCAGCTTTGTGGAAAAAGAATGGGCCTTTATTTCTCTGGGGAATGAATTCCTGTGGCATCTGATGCTCTAGGACCATAAAGAGCTCACTAAAGCTTGTAACTTTACTGCTGTCCTACCTCACTAAACTGCCCTATGGAATAATTCCT

		CAAGCTGTTTTCCTTTTCGTCATCTTTGGCGAAATAGGGGAAGAGCCTCTC
Pgl_GLEAN_10010905 ("10010905p")	Pearl millet PLAT/LH2 domain-containing lipoxygenase family protein Encodes lipoxygenase5 (LOX5). LOX5 activity in roots facilitates green peach aphid colonization of Arabidopsis foliage by promoting green peach aphid feeding from sieve element and water consumption from xylem. LOX5; FUNCTIONS IN: oxidoreductase activity, acting on single donors with incorporation of molecular oxygen, incorporation of two atoms of oxygen, lipoxygenase activity, iron ion binding, metal ion binding	GGCACAGGTGCTGGCGTGCCCCCGTAGTCTCGGTTCGTAATACCACCCGGGACTAAAGGAGCCTTTAGTCCCGGTTGGTTCAAGCTGGACTAGGGGGGCTTTTGTCTCTAAACTTTAGTCCTGGTTGGACTTGAGAGACCTAAACCCCTTTCCAACCGGGACTAAAGCCCCTCTCCCTACCAGTGACAGGTGGGTTCTAGTGAATGAATAAGAGGAGTAGAGGATGGAAGTAGAGTGTACGCGTGCAGTAGGATGTTCTTTTTGAGGTATATTTAGAGGACATCTAACATATGTGTTGGATATAGATTTGGGTTATTACTTATTATTACTTGTCAGGCTGTCACCAGCCACCACTGATTCTTGAAACCAAATCCATCAGATTCCCAAGAGTGCCAAAGGGAATCTCTCCTTCTGCATCCCTCTATATAGTGCCGATCGAAACACAAAGCACATTGCCCTCAGTGTCCCATACGGAAAACCGGCCCCCATCGAGCGTGGAC

[a]The W-box consensus sequence TTGAC(C/T) or its complementary sequence (A/G)GTCAA is shown in red with a background color. The W-box-like sequence (C/T)GAC followed by 0-3 repeats of T or its complementary sequence is shown in red and underlined.

Supplementary Table 4. Primers used for qRT-PCR.

**Primer name**	**Sequence (5 ´to 3´)**	**Target gene**
RD29A_Fw	GTTACTGATCCCACCAAAGAAGA	*RD29A* (AT5G52310)
RD29A_Rv	GGAGACTCATCAGTCACTTCCA	
DREB2A_Fw	AAGGGTCGAAGAAGGGTTGT	*DREB2A* (AT5G05410)
DREB2A_Rv	CGAGCCAAAGGACCATACAT	
RD29B_Fw	AAAAGAGAGGCACCGACTCA	*RD29B* (AT5G52300)
RD29B_Rv	CCGTTGACCACCGAGATAGT	
GAPDH_Fw	TTGGTGACAACAGGTCAAGCA	*GAPDH* (AT1G13440)
GAPDH_Rv	AAACTTGTCGCTCAATGCAATC	
PgWRKY74_Fw	CCGAGCTACGTTGTGACCAT	*PgWRKY74*
PgWRKY74_Rv	CGTCTTGGGTGGCGTAGTAG	
